# Proteome and Peptidome of Human Acquired Enamel Pellicle on Deciduous Teeth

**DOI:** 10.3390/ijms14010920

**Published:** 2013-01-07

**Authors:** Jason N. Zimmerman, William Custodio, Sahza Hatibovic-Kofman, Young Ho Lee, Yizhi Xiao, Walter L. Siqueira

**Affiliations:** Schulich School of Medicine & Dentistry, Western University, London, ON N5A6C1, Canada; E-Mails: jzimmerman2014@dents.uwo.ca (J.N.Z.); wcustodi@uwo.ca (W.C.); sahza.kofman@schulich.uwo.ca (S.H.-K.); ylee347@uwo.ca (Y.H.L.); yxiao32@uwo.ca (Y.X.)

**Keywords:** acquired enamel pellicle, saliva, proteomics, primary teeth, proteins, oral, LC-MS/MS

## Abstract

Understanding the composition and structure of the acquired enamel pellicle (AEP) has been a major goal in oral biology. Our lab has conducted studies on the composition of AEP formed on permanent enamel. The exhaustive exploration has provided a comprehensive identification of more than 100 proteins from AEP formed on permanent enamel. The AEP formed on deciduous enamel has not been subjected to the same biochemical characterization scrutiny as that of permanent enamel, despite the fact that deciduous enamel is structurally different from permanent enamel. We hypothesized that the AEP proteome and peptidome formed on deciduous enamel may also be composed of unique proteins, some of which may not be common with AEP of permanent enamel explored previously. Pellicle material was collected from 10 children (aged 18–54 months) and subjected to mass spectrometry analysis. A total of 76 pellicle proteins were identified from the deciduous pellicle proteome. In addition, 38 natural occurring AEP peptides were identified from 10 proteins, suggesting that primary AEP proteome/peptidome presents a unique proteome composition. This is the first study to provide a comprehensive investigation of *in vivo* AEP formed on deciduous enamel.

## 1. Introduction

The relationship between human saliva and dental enamel is a complex one, including that of the formation of the acquired enamel pellicle (AEP). AEP is a thin layer that is formed predominantly from salivary proteins and their products by selective adsorption onto the enamel surface [1]. AEP consists predominantly of salivary proteins and peptides, but also includes non-salivary derived proteins, carbohydrates and lipids. This creates a protective interface between the tooth surface and the oral environment, and acts as a selectively permeable barrier that regulates the demineralization and remineralization processes of enamel [2]. Furthermore, AEP influences the composition of the micro flora that inhabits the tooth surface [3].

Improvements in sensitive proteomic methodologies have provided new opportunities for the characterization of very-low-abundance biological samples, including AEP. New technologies like mass spectrometry have resulted in the characterization of the proteome of human permanent teeth pellicle [4]. From the permanent AEP, 130 proteins have been identified and characterized according to origin, putative biological function, and potential role in AEP structure. When these proteins were categorized based on their possible role in AEP development, three major groups were classified. These groups were comprised of proteins that bind to calcium ions, proteins with a high affinity for phosphate ions, and proteins that have been shown to have interactions with other proteins. AEP proteins have also been classified according to putative biological functions such as inflammatory response, immune defense, antimicrobial, and remineralization capacity.

Data collected on the permanent AEP peptidome revealed the presence of multiple protein fragments from histatins, statherin, and acidic PRPs [5,6]. This can be related to the fact that these proteins adsorb strongly to the hydroxyapatite of enamel, and are readily cleaved by proteolytic enzymes found in the oral environment [6,7]. In addition, these proteins present a high abundance in whole saliva and consequently it is suggested a high abundance in the *in vivo* AEP in intact protein structure and peptides fragments. The discovery of many peptides in AEP is significant because it is well known that many salivary proteins have functional domains that when isolated, not only maintain but in some cases exceed the activities of the native intact parental protein [7]. Therefore, AEP is a complex biological structure comprised of specific salivary protein fragments and intact native proteins.

Extensive research has already been done regarding the protein composition of AEP formed on permanent teeth, while studies on the proteome of the AEP of deciduous teeth have not been reported [4,6,8,9]. This is interesting since the enamel of deciduous teeth, the surface onto which the AEP is selectively adsorbed, is structurally different from the enamel of permanent teeth [10].

Enamel is made of enamel crystals, which are in turn made of enamel rods. In the enamel of permanent teeth, rods are oriented perpendicular to the enamel surface. In deciduous teeth, the enamel rods are organized obliquely to the enamel surface at angles that vary from 40 to 60 degrees [11]. Also, the crystallite deviation in enamel of deciduous teeth is significantly less than that seen in permanent teeth. Therefore, the oblique organization of enamel rods and their significantly smaller crystallite deviation result in a clear and quantifiable difference between the surface and subsurface crystallite orientation in deciduous teeth [11]. This has not been found in permanent enamel where the crystallite organization in surface enamel is fairly analogous to what is observed in sub-surface enamel [11]. In addition, primary enamel is softer, has a higher porosity, and is less elastic than permanent enamel [12].

The dental enamel can be divided into prismatic and prismless layers, each histologically different from each other. The crystal organization of the prismless enamel, the outer layer of the dental surface, differs from the permanent to deciduous teeth. In permanent teeth, the prismless enamel is comprised of two different stratums. One type occurs as a continuous band similar to that seen in deciduous dentition. The other type of prismless enamel, exclusively seen in permanent teeth, has its crystal structure arranged as a scale-like layer [10].

There are also differences in amino acid composition between the AEPs of permanent and deciduous teeth. Although a generally similar pattern in the amount of the amino acids has been seen, the amino acids glycine, serine, and tyrosine were reported in statistically significantly different quantities in the two types of AEP [13]. This suggests that the pellicles may have different protein compositions.

In the present study, we investigated the proteome and peptidome of human AEP from deciduous teeth *in vivo*. We hypothesize that the proteome and peptidome of human deciduous *in vivo* AEP will present a unique protein profile.

## 2. Results and Discussion

**Figure 1:**
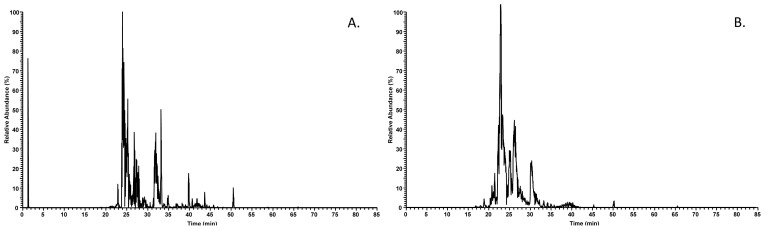
Examples of base-peak chromatograms. (**A**) Base-peak chromatogram of a pooled acquired enamel pellicle (AEP) sample. Peptides generated by trypsinization were loaded on a nanoscale RP-HPLC column, and eluted in a gradient from 5% to 55% buffer B in 65 min. (**B**) Base-peak chromatogram of a pooled AEP sample. Peptides below than 10 kDa were separated by centrifugal filtration and loaded on a nanoscale RP-HPLC column, and eluted in a gradient from 5% to 55% buffer B in 65 min. On the y-axis, the peptide ion peak intensity is plotted.

The base-peak chromatogram for reversed-phase chromatography monitored by the mass spectrometer represents the intensity of all peptide ions in the sample in a single scan after trypsin digestion for proteome analysis (Figure 1A), and after centrifugal filtration with MWCO < 10 kDa to maintain AEP occurring peptides for peptidome analysis (Figure 1B). It is clear that in both base-peak chromatograms, the majority of the peptides were eluted between 20 and 35 min. This observation is in agreement with our previous *in vivo* AEP investigation for permanent teeth where the majority of the peptides were eluted with a similar retention time [6,10]. In addition, it is easy to note that the base-peak chromatogram generated after trypsin digestion presents a higher peptide complexity than the base-peak chromatogram from natural occurring AEP peptides with MW < 10 kDa. This complexity reflects in the number of protein and peptides identified in these two different proteomic approaches.

### 2.1. AEP Proteome

A total of 76 different pellicle proteins were identified from the *in vivo* deciduous pellicle proteome (Table 1). The most abundant salivary proteins based on classical biochemical studies [14–16] that are commonplace in permanent AEP were also present in the deciduous AEP. These proteins include α-amylase, acidic PRPs, histatins, cystatins, statherin, and mucins [4]. After analyzing the proteome of the deciduous AEP, interesting patterns emerge.

**Figure 2:**
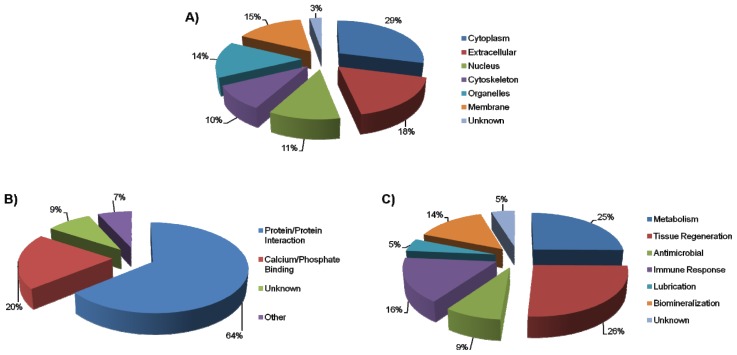
Classification of the *in vivo* deciduous AEP proteins according to (**A**) origin, (**B**) molecular interaction, (**C**) biological function. The area of each sector is directly proportional to the number of proteins in the deciduous AEP proteome that corresponds to each specific group.

It has been well established that the AEP is formed by the selective adsorption of salivary proteins and their products to the enamel surface [1,6]. The proteins comprising the deciduous AEP proteome can be grouped according to their origin (Figure 2A). It was not surprising to see that a large number of identified proteins were extracellular in origin. This is to be expected because many of the AEP proteins are secreted from the major and minor salivary glands [17,18]. In total, those salivary glands are responsible for more than 90% of the volume of saliva produced [17]. On the other hand, it is also notable that there were many proteins that originated from the cell cytoplasm. This is most likely due to the high turnover rate of oral mucosa cells [19].

When the identified pellicle proteins were analyzed according to their role in AEP structure formation or molecular interaction, the proteins can be segregated into four main groups (Figure 2B). The first group comprises proteins that potentially have the ability to bind to calcium and phosphate ions. Examples of these proteins include cytoplasm origin proteins such as calmodulin, and also secreted salivary gland proteins such as statherin and histatin 1. Due the mineral composition of the enamel, mainly composed of hydroxyapatite which is a crystal formed of calcium phosphate, these proteins have the potential to interact directly with the calcium and phosphate ions of the enamel surface. They can therefore be considered pellicle precursor proteins, forming the primary protein layer by adsorbing directly onto the enamel surface. Approximately 20% of the identified proteins from this study showed the ability to interact with calcium and phosphate ions. In addition, salivary proteins that are well known to adsorb to dental enamel [20], such as carbonic anhydrase VI (CA-VI), have not been identified in the deciduous AEP. The non-identification of such proteins can be a result of the highly selectiveness of the adsorption process or the low enzymatic content in the deciduous AEP proteome (2 h AEP formation).

The second categorized group (64%, Figure 2B) consists of proteins that are able to interact with other proteins. An example is MUC5A, which has been known to form complexes with many other salivary proteins, including salivary α-amylase which was also detected in this study as a component of the *in vivo* deciduous AEP. Proteins of this group potentially form the successive protein layers in the AEP by interacting with the pellicle precursor proteins that are adsorbed directly onto the enamel surface. The third group consists of proteins that have an unknown mode of molecular interaction, which encompassed around 9% of the identified proteins. It is interesting to note that the number of proteins with unknown molecular interaction is now fewer than in previous studies that discovered pellicle proteins with an unknown mechanism of molecular interaction [4]. This is possibly due to the advancement in proteomics and the ever-increasing protein database information, and their biological functions and interactions. Every year more proteins are being discovered along with how they interact with their environment, leaving far fewer gaps in our knowledge about how proteins could behave when in physiological environment. The last group, consisting of roughly 7% of the identified proteins, had mechanisms of molecular interaction outside of the above groups. Examples of these proteins include histone H2B and 60S acidic ribosomal protein P2, which bind to DNA and RNA respectively. It is also interesting to note that many of the discovered deciduous AEP proteins have multiple modes of interaction, for example lactoferrin-C which has the ability to interact with both calcium ions and other proteins.

Thirdly, the newly discovered pellicle proteins of the deciduous AEP proteome may be categorized based on their biological function within the AEP such as metabolism, tissue regeneration, antimicrobial activity, immune response, lubrication, and biomineralization (Figure 2C). Due to the complex and dynamic environment present in the oral cavity, all those biological functions are closely related to essential biological functions carried out by the AEP structure. It is important to highlight that an overlap is seen in proteins that are involved in biomineralization processes and those that have a high affinity for the calcium and phosphate ions of the enamel surface, like the statherin or histatin proteins [21,22]. Approximately 14% of the identified proteins are involved in biomineralization processes, which are important in maintaining the homeostasis of the enamel and consequently, the teeth themselves and oral health. Additional proteins discovered in the deciduous AEP are involved in the immune or inflammatory response, or display antimicrobial properties. Both of these biological functions are imperative to allow for host defense against oral pathogens. Some identified proteins exhibiting these functions include myeloperoxidase, cystatin, and lysozyme. This group of proteins along with those involved in tissue regeneration account for around 52% of the identified pellicle proteins in total, and has the potential to be protective against oral infective pathologies like periodontal disease. Interestingly, there were proteins found, like myeloperoxidase, which maintain their defensive properties upon adsorption to the hydroxyapatite of enamel [23]. Along with myeloperoxidase, albumin and other identified pellicle proteins are known to have fluctuating concentrations in the gingival crevicular fluid, an exudate present between the periodontal tissue and the tooth, and in the AEP, depending on the degree of inflammatory response as a result of periodontal disease [24]. Therefore, these proteins may signify crucial biomarkers for oral inflammatory infections [24].

Even considering the limitations regarding the comparison among proteins identified in different proteomic studies, our newly discovered *in vivo* deciduous pellicle had 42% of our proteins in common with previous studies on *in vivo* permanent teeth [4,9]. Since the AEP is formed by selective adsorption onto the enamel surface and primary enamel is structurally different from permanent enamel, the primary and permanent AEPs do not share the majority of their proteins. This proteome difference is attributed to the enamel surface properties and mineral content, where the mineral content of primary enamel is 81 to 94 wt % while permanent enamel has around 97 wt % mineral content [25,26]. Therefore, the selective adsorption of salivary proteins onto the enamel surface could be affected by this significant difference in surface mineralization. In addition, it is well defined that not all salivary proteins have the ability to adsorb and stay on the enamel surface [1,20]. Previous studies demonstrated that only less than 5% of the total identified salivary proteome [17,18,27–31] is able to bind to the enamel surface forming the AEP [20]. Moreover surface change composition, for example with incorporation of fluoride on the surface of the enamel can modulate qualitatively and quantitative the adsorption of specific salivary proteins on the enamel surface [8]. All these statement reinforce the importance of the AEP as a unique protein film in relation to structure and composition.

### 2.2. AEP Peptidome

The molecular weight limitation for a direct characterization by MS/MS is an obstacle not easily overcome. Therefore, the restriction to the analysis of the less than 10 kDa molecules provided a more realistic insight into the composition of at least small pellicle components than endeavors into the full spectrum of deciduous pellicle proteins dependent on tryptic fragmentation prior to MS analysis. Despite the limitations in size of the pellicle protein/peptides studied, these small pellicle constituents did represent a very significant portion of the deciduous pellicle proteome.

**Figure 3:**
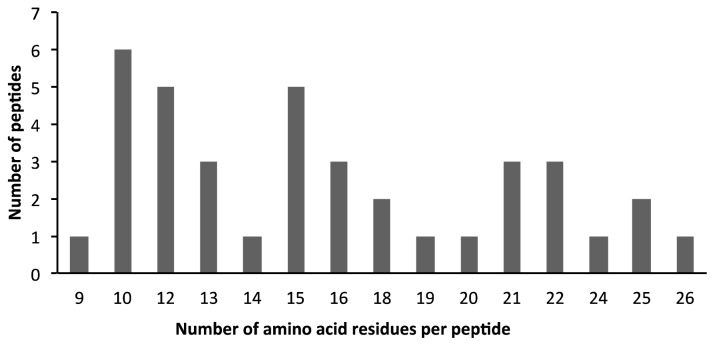
Size distribution of AEP peptides according to the number of amino acid residues per peptide.

After the centrifugal filtration of the AEP protein/peptide material with a MWCO membrane of 10 kDa, the MS analysis of the deciduous teeth peptidome resulted in the identification of 38 natural occurring peptides with molecular weights ranging from 956 to 2621 Da from 10 different proteins (Table 2). Peptides characteristics such as number of amino acid residues, XCorr, MS/MS charge state, molecular weight, isoelectric point and net charge at pH 7.0 are showed in Table 2. It was not surprising that the majority of the peptidome consisted of peptides with 10 to 13 amino acid residues (Figure 3). This is in accordance with past studies of the AEP peptidome of permanent teeth [6]. The major contributors to the deciduous AEP peptidome are aPRPs, bPRPs, histatins and statherin. This is consistent with two known properties of these proteins: they adsorb strongly to the enamel surface and they are readily cleaved by proteolytic enzymes present in the oral environment [32–34].

Similarly with the AEP peptidome of permanent teeth, 51% of the peptides originated from *C*-terminal domains of the pellicle precursor proteins while only 22% of the peptides originated from *N*-terminal domains (Table 2). Another similarity with the permanent AEP peptidome was related to the large fraction of hydrophobic peptides that were encountered, indicating that ionic interactions with the enamel surface are not the only driving force for AEP formation (Table 2) [6]. In addition, more than two-thirds of the identified naturally occurring AEP peptides exhibited a positive net charge (Table 2). This data reinforces the concept that the AEP peptides and proteins characterized in this study constitute a mixture of components directly adsorbing to hydroxyapatite, and components interacting with other pellicle constituents. The characteristics of hydrophobicity and net charge are equally important for peptide-peptide or protein-peptide interactions, leading to cluster structures typical in the AEP development.

**Figure 4:**
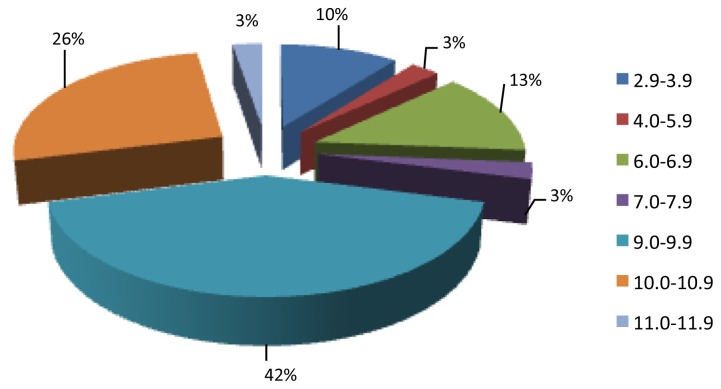
Isoelectric point range distribution of AEP natural occurring peptides at pH 7.0.

The oral cavity presents a pH around 6.9. Thus, the pellicle peptides were grouped according to their isoelectric point at pH 7.0 (Figure 4). It is interesting to note that approximately 26% exhibited isoelectric points below 6.9. This means that they have a negative charge at a neutral pH and therefore, in the oral environment. Consequently this group of natural occurring peptides has the potential to directly bind to the enamel surface. On the other hand, more than 71% of the identified peptides exhibited isoelectric points between 9.0 and 11.9 (Figure 4). These peptides present a unique characteristic when compared with the salivary peptidome where the majority of the peptides displayed isoelectric points below 6.9 [35]. Again, our data confirms that the formation of AEP is a selective process where specific proteins/peptides adsorbs to the enamel surface [1] while other salivary proteins/peptides are not able to adsorb onto the enamel surface.

When the cleavage sites were thoroughly analyzed, a trend was seen that 24% of the all identified peptides were cleaved after KPQ amino acid residues (Table 2). Furthermore, 8% of the proteins were cleaved after PQP amino acid residues. These two cleavage sites are known to be preferred sites for salivary proteases [35] and consequently helping in the formation of *in vivo* AEP. However, the exploration of the proteolytic mechanisms needs to be addressed in the future to better characterize whether this phenomenon is occurring in the saliva itself or after binding to the enamel surface.

## 3. Experimental Section

### 3.1. Human Subjects

AEP was obtained from ten healthy children (male and female) with no dental caries active, ranging in age from 18–54 months. The subjects did not exhibit any oral condition that could affect oral fluid composition. AEP collection protocols were approved by the Research Human Ethics Board of The University Western University (review number 16181E). Written informed consent was acquired from the parents or guardians of all subjects in this study.

### 3.2. AEP Collection

The procedure used for *in vivo* AEP collection was carried out as described previously [6]. Samples were always collected in the morning to avoid circadian effects on pellicle composition. Briefly, each donor was subjected to a dental prophylaxis treatment employing coarse pumice containing no additives. AEP was then allowed to form on the enamel surfaces over a 2-h period in order to have a pellicle comprised by both precursor proteins and proteins clusters. During this time span, the participants were asked to refrain from any consumption of food or beverages, other than water. After 2 h, teeth from each quadrant were isolated with cotton rolls, washed with water using the dental unit’s built-in spray gun, and dried by air.

For the actual removal of AEP material from the enamel surface, collection strips of 0.5 cm × 1.0 cm (electrode wick filter paper, Bio-Rad, Hercules, CA, USA) pre-soaked in 3% citric acid was folded so that one half could be held using a dental forceps (Hu-Friedy, Chicago, IL, USA) and the other half could be brought in contact with the tooth surface. To avoid any contamination emanating from the gingival margin, only the coronal two thirds of the labial/buccal surfaces were swabbed. One collection strip was used per quadrant, starting with the buccal area of the central incisor and ending with the buccal surface of the first molar. The collection was carried out in both dental arches. A total of four collection strips from each participant were obtained per collection and placed into a polypropylene microcentrifuge tube. The collection strips were then kept frozen at −80 °C until used.

### 3.3. AEP Elution by Sonication

To extract the AEP proteins from the collection strips, 200 μL of 50 mM ammonium bicarbonate, pH 7.8 was added to each polypropylene microcentrifuge tube, containing four collection strips from each subject. Each microcentrifuge tube was then sonicated for 1 min, and the recovered solution was then collected and placed into a new microcentrifuge tube for each subject. This procedure was repeated for a total of 4 times. The extracted solution was then centrifuged at 14,000× *g* for 15 min and the supernatant was extracted. This centrifugal procedure was carried out to prevent the debris from the collection strip that could be released into the solution during sonication step. The supernatant was dried using a rotary evaporator (Eppendorf, Parkway, NY, USA), and then resuspended in 100 μL of distilled water. Micro bicinchoninic acid (Micro BCA) assay was carried out to determine the total protein concentration of the extracted solution from each subject.

### 3.4. Deciduous AEP Proteome

The equivalent of 2 μg of AEP protein was taken from each of the 10 samples and placed into one polypropylene microcentrifuge tube, creating a pool of 20 μg. All samples were dried using a rotary evaporator and stored at 4 °C until they were needed for further experimentation.

### 3.5. In-Solution Digestion

Dried samples were resuspended in 50 μL of 4 M urea, 10 Mm DTT and 50 mM ammonium bicarbonate at pH 7.8 and incubated for 1 hour at room temperature. Afterwards, 150 μL of 50 mM ammonium bicarbonate was added to the samples, followed by 2% (*w*/*w*) trypsin (Promega, Madison, WI, USA). The sample was then allowed to incubate overnight at 37 °C. Finally, the samples were dried in a rotary evaporator, de-salted by C-18 ZipTip^®^ Pipette Tips (Millipore, Billerica, MA, USA), and subjected to mass spectrometry.

### 3.6. Deciduous AEP Peptidome

From the initial 10 eluted samples, a total of 4 μg of AEP protein from each subject was placed into a microcentrifuge tube, creating a single sample containing 40 μg of AEP protein. The sample was filtered by centrifugal filtration using a 10 kDa molecular weight cut-off (MWCO) membrane (Pall Life Sciences, Ann Arbor, MI, USA). The eluted AEP was centrifuged for 10 min at 14,000× *g* using a refrigerated eppendorf table-top centrifuge (Eppendorf, Parkway, NY, USA). The filtrate containing the proteins/peptides with molecular weights below 10 kDa was collected, dried, and subjected to MS analysis.

### 3.7. LC-ESI-MS/MS Analyses

Mass spectrometric analyses were carried out with a LTQ-Velos (Thermo Scientific, San Jose, CA, USA) which allows for in-line liquid chromatography with the capillary fused silica column (column length 10 mm, column ID 75 μm) packed in-house using C-18 resin of 3μm spherical beads and 100 Å pores size (Michrom BioResources, Auburn, CA, USA) linked to the mass spectrometer using an electrospray ionization in a survey scan in the range of *m*/*z* values 390–2000 tandem MS/MS. A dynamic exclusion criterion was established as a repeat count of 1 and a repeat duration of 30 s. All samples were dried by rotary evaporator and re-suspended in 15 μL of 97.5% H_2_O/2.4% acetonitrile/0.1% formic acid and then subjected to reversed-phase LC-ESI-MS/MS. The nano-flow reversed-phase HPLC was developed with linear 65-minute gradient ranging from 5% to 55% of solvent B (97.5% acetonitrile, 0.1% formic acid) at a flow rate of 200 nL/min with a maximum pressure of 280 bar. Electrospray voltage and the temperature of the ion transfer capillary were 1.8 kV and 250 °C respectively. Each survey scan (MS) was followed by automated sequential selection of seven peptides for CID, with dynamic exclusion of the previously selected ions.

### 3.8. Peptide and Protein Identification

For proteome and peptidome analysis, the obtained MS/MS spectra were searched against human protein databases (Swiss Prot and TrEMBL, Swiss Institute of Bioinformatics, Geneva, Switzerland, http://ca.expasy.org/sprot/) using SEQUEST and Percolator algorithms in Proteome Discoverer 1.3 software (Thermo Scientific, San Jose, CA, USA). A maximum of two miscleavages were allowed; Carbamydomethylation of cysteine; phosphorylation of serine, threonine and tyrosine; and oxidation of methionine were included as dynamic modification. While for proteome analysis, trypsin specific cleavage site was considered, peptidome analysis was carried out with no specify fragmentation search. Search results were filtered for a False Discovery Rate of 1% employing a decoy search strategy utilizing a reverse database. A total of three mass spectrometric runs were carried out in each condition.

### 3.9. Protein Annotations

The identified proteins were classified and assigned by origin, molecular interaction and biological function using three web-based applications: Babelomics database (http://babelomics.bioinfo.cipf.es/index.html), AmiGO database (http://amigo.geneontology.org/cgi-bin/amigo/go.cgi?advanced_query=yes), and Swiss protein database (http://ca.expasy.org). In addition, natural occurring peptides were characterized using a web-tool (http://pepcalc.com/ppc.php).

## 4. Conclusions

Recognizing the limitations of the present study, the primary AEP proteome/peptidome demonstrated different trends when compared with the permanent AEP proteome/peptidome from previous studies. This study emphasizes the importance of investigating the physiological conditions of the AEP on different enamel substrates (permanent and primary teeth). As a consequence of different AEP composition between primary and permanent teeth, clinically the mechanism behind the initiation of oral pathologies related to this protein film could be different.

## Supplementary Information



## Figures and Tables

**Table 1:** Identified proteins from *in vivo* AEP formed on deciduous enamel.

Accession #	Protein Name
B7ZMD7	Amylase, alpha 1A (Salivary) b, h, i, l, n, q
P02814	Submaxillary gland androgen-regulated protein 3B b, k, r
F6KPG5	Albumin (Fragment) a, b, e, f, h, i, l, m, q
P02533	Keratin, Cytoskeletal 14 d, h, m
P08779	Keratin, Cytoskeletal 16 a, d, e, h, m
B4DRW1	Keratin, Cytoskeletal 4 a, d, h, m
A1A4E9	Keratin, cytoskeletal 13 a, d, e, h, m
P02812	Basic salivary proline-rich protein 2 a, d, h, l, o, p
H6VRF8	Keratin 1^d^, ^h^, ^l^, ^o^
P02808	Statherin b, f, h, i, p, q
B5BU38	Annexin a, b, c, d, e, f, h, i, l, m, o, q
P07476	Involucrin d, f, h, l, m
B4DWU6	cDNA FLJ51361, highly similar to Keratin, type II cytoskeletal 6A d, h, m
B4DVQ0	cDNA FLJ58286, highly similar to Actin, cytoplasmic 2 a, f, h, m
P35908	Keratin, type II cytoskeletal 2 epidermal a, b, h, m
C9JEV0	Zinc-alpha-2-glycoprotein b, f, h, o
P02810	Salivary acidic proline-rich phosphoprotein ½ b, f, h, i, q
P06702	Calgranulin B a, b, c, d, e, f, h, i, n, o, q
P04280	Basic salivary proline-rich protein 1 b, j, l, p
P13647	Keratin, type II cytoskeletal 5 a, d, e, f, h, m
P01040	Cystatin-A a, c, e, h, n
P01834	Ig kappa chain C region b, f, h, o
P0CG05	Ig lambda-2 chain C regions a, d, h, o
B7Z4X2	Lactoferroxin-C b, i, l, q
P61626	Lysozyme b, h, n, o
B2R4M6	highly similar to Homo sapiens S100 calcium binding protein A9 a, c, d, i, l, m, o, q
P15515	Histatin 1 c, h, n, q
P12273	Prolactin-inducible protein b, h, r
B1AN48	Small proline-rich protein 3 (Fragment) a, h, l, m
Q01546	Keratin, type II cytoskeletal 2 oral d, h, m
P98088	Mucin-5AC (Fragments) a, b, e, h, m, n, p, q
E7EQV5	Actin, alpha skeletal muscle a, d, h, m
H0YKS4	Annexin (Fragment) a, f, h, i, l, m, o, q
P01876	Ig alpha-1 chain C region b, f, h, n, o
F8VV32	Lysozyme C b, h, n, o
O60744	Thioredoxin delta 3 (Fragment) a, b, c, e, h, l, m, o
P10163	Basic salivary proline-rich protein 4 b, j, l, p
B4DIL4	cDNA FLJ50166, highly similar to Dedicator of cytokinesis protein 6 a, h, o
B7Z7R8	cDNA FLJ55622, highly similar to Multimerin-1 a, b, e, h, o
H0Y6K7	Probable E3 ubiquitin-protein ligase HERC4 (Fragment) a, f, j, l, m
P31947	14-3-3 protein sigma OS=Homo sapiens a, b, c, h, l, m
P07108	Acyl-CoA-binding protein a, e, h, l
D6RCA8	Annexin (Fragment) b, f, h, i, l, m, o, q
H0YMD9	Annexin A2 (Fragment) g, h, i, l, m, o, q
Q86VF0	Beta-globin (Fragment) a, b, h, i, l, m, o, q
A0M8Q9	C1 segment protein (Fragment) g, h, l, o
P27482	Calmodulin-like protein 3 a, i, q
B4DWR5	highly similar to Involucrin a, f, j, r
B4DGW2	highly similar to Rho guanine nucleotide exchange factor 12 d, h, m
A8K5I6	highly similar to Homo sapiens cornulin (CRNN) a, b, c, d, e, f, i, m, q
P04080	Cystatin B b, h, n
P01036	Cystatin S a, b, f, h, n
Q9UGM3	Deleted in malignant brain tumors 1 protein b, h, i, n, q
G3V1R1	HCG26567, isoform CRA_c a, c, d, e, f, j, p
F8WE04	Heat shock protein beta-1 a, b, e, f, h, l
E5RG22	Hemofiltrate peptide HF7665 (Fragment) b, h, m
A8K9J7	Histone H2B g, k, m
H0Y3I2	Lactoperoxidase a, b, c, e, i, n, q
P05164	Myeloperoxidase a, b, c, e, i, n, q
C9J4S4	Ras-related protein Rab-7a a, e, h, l, m
E9PBV3	Suprabasin a, e, j, r
H0YDD8	60S acidic ribosomal protein P2 (Fragment) a, e, k, l, m
Q2MD48	B-cell linker protein (Fragment) a, f, h, o
F8WBR5	Calmodulin g, i, q
Q9Y6Y1	Calmodulin-binding transcription activator 1 a, c, h, k, l
B4DT21	cDNA FLJ50202, highly similar to Interleukin-12 receptor beta-2 chain f, h, l, m
H0YCD2	DNA polymerase subunit gamma-1 (Fragment) a, e, f, h, k, l, m
G3V1M9	HCG26567, isoform CRA_b b, j, l
O60393	Homeobox protein NOBOX c, f, k, l, m
C9J0S5	Lactoferroxin-C (Fragment) a, b, h, i, l, o, q
E9PNX2	Neuronal acetylcholine receptor subunit alpha-10 f, h, m
Q59H51	Pleckstrin homology domain-containing protein family A member 4 variant (Fragment) a, f, h, r
B7ZW15	Putative uncharacterized protein a, h, l
F5H4J4	Runt-related transcription factor 3 a, c, e, h, k, l, m
Q9BVH8	VWA5B2 protein (Fragment) g, h, r

a cytoplasm origin;

b extracellular origin;

c nucleus origin;

d cytoskeleton origin;

e organelles origin;

f membrane origin;

g unknown protein origin;

h protein/protein interaction;

i calcium/phosphate binding;

j unknown molecular interaction;

k other molecular interaction;

l metabolism;

m tissue regeneration;

n antimicrobial;

o immune response;

p lubrication;

q biomineralization;

r unknown biological function.

**Table 2:** Identified naturally occurring peptides from *in vivo* AEP originating from deciduous enamel *.

Accession	Protein name	Peptide sequence	# AA	XCorr	MS/MS charge	MW (Da)	pI	Net charge at pH 7
P02808	Statherin	FGYGYGPYQPVPEQP	15	3.73	2	1699.59	3.3	−1
		YGPYQPVPEQPLYPQP	16	3.25	2	1874.25	3.3	−1
		YQPVPEQPLYPQP	13	3.24	2	1556.12	3.3	−1

P02810	Salivary acidic proline-rich phosphoprotein 1/2	GHQQGPPPPPPGKPQ	15	3.17	3	1517.01	10.1	1.1
		GPPPQGGRPQ	10	2.60	2	989.95	11	1
		GPPPQGGRPQGPPQGQSPQ	19	4.05	2	1867.39	11	1
		GPPQQGGHPRPP	12	2.65	2	1224.51	11	1.1
		GRPQGPPQGQSPQ	13	3.32	2	1334.13	11	1
		QGPPQQGGHQQGPPPPPPGKPQ	22	4.54	3	2211.75	10.1	1.1

P02812	Basic salivary proline-rich protein 2	GNQPQGPPPP	10	2.53	2	988.30	6.01	0
		GPPPPGKPQGPPPQ	14	4.01	2	1351.15	10.1	1
		GPPSPPGKPQ	10	2.59	2	961.79	10.1	1
		KPQGPPPPGKPQGPPPQGDK	20	5.25	3	2004.77	10.3	2
		KPQGPPPPGKPQGPPPQGDNK	21	3.40	3	2119.08	10.3	2
		PGKPQGPPPQGGSKSRSARSP	21	2.71	2	2073.93	12.4	4
		PGKPQGPPPQGGSKSRSARSPPGKP	25	2.74	2	2452.99	12.4	5
		PGKPQGPPPQGGSKSRSSRSPPGK	24	3.14	2	2452.99	12.4	5

P02814	Submaxillary gland androgenregulated protein 3	FGPGFVPPPPPPPYGPGR	18	2.61	2	1834.38	9.8	1
		FVPPPPPPPYGPG	13	3.11	2	1318.79	5.9	0
		GPGIFPPPPPQP	12	2.95	2	1202.34	6	0
		GPLAPPQPFGPGFVPPPPPPPYGPGR	26	3.27	3	2591.58	9.8	1

P04080	Cystatin-B	SQVVAGTNYFIK	12	4.21	2	1327.52	9.7	1

P04280	Basic salivary proline-rich protein 1	GGNKPQGPPPPPGKPQ	16	3.37	3	1553.24	10.6	2
		GNKPQGPPPP	10	2.63	2	987.61	10.1	1
		GNKPQGPPPPGKPQGPPPQGDK	22	3.73	3	2175.93	10.3	2
		GNKPQGPPPPPGKPQ	15	3.14	3	1496.85	10.6	2
		GNQPQGPPPP	10	2.53	2	988.30	6	0
		GPPPPGKPQGPPAQG	15	2.59	2	1381.12	10	1
		KPQGPPPPGKPQGPPAQGGSK	21	4.01	3	2007.97	10.8	3
		PQGGNKPQGPPPPPGKPQ	18	2.78	2	1779.23	10.6	2

P06702	S100-A9	NIETIINTFHQYSVK	15	4.61	2	1807.65	7.7	0.1
		NIETIINTFHQY	12	3.27	2	1492.53	5.1	−0.9

P10163	Basic salivary proline-rich protein 4 allele S	GNKPQGPPPP	10	2.63	2	987.61	10	1
		LISGKPEGR	9	2.84	2	956.46	10	1

P15515	Histatin 1	EFPFYGDYGSNYLYDN	16	5.24	2	1964.32	2.8	−3
		REFPFYGDYGSN	12	2.88	2	1452.20	4	−1

Q8TAX7	Mucin-7	PVNSPAPQDTTAAPPTPSATTP	22	2.70	2	2277.10	3.1	−1
		TSSSVATLAPVNSPAPQDTTAAPPT	25	3.73	3	2621.38	3.1	−1

* MS/MS spectra with labeled b and y ions are provided as supplemental data. MS/MS spectra are demonstrated according to the appearance order in the Table 2.
