# Supplementary material for: Proteome and Peptidome of Human Acquired Enamel Pellicle on Deciduous Teeth
Source: Int J Mol Sci. 2013 Jan 7;14(1):920–34. doi: 10.3390/ijms14010920 (PMC3565298; doi:10.3390/ijms14010920)
Supplement: Supplementary file 1 [file ijms-14-00920-s001.pdf]

Supplementary Information

Figure S1. Identified MS/MS spectra for naturally occurring AEP peptides for statherin (P02808).

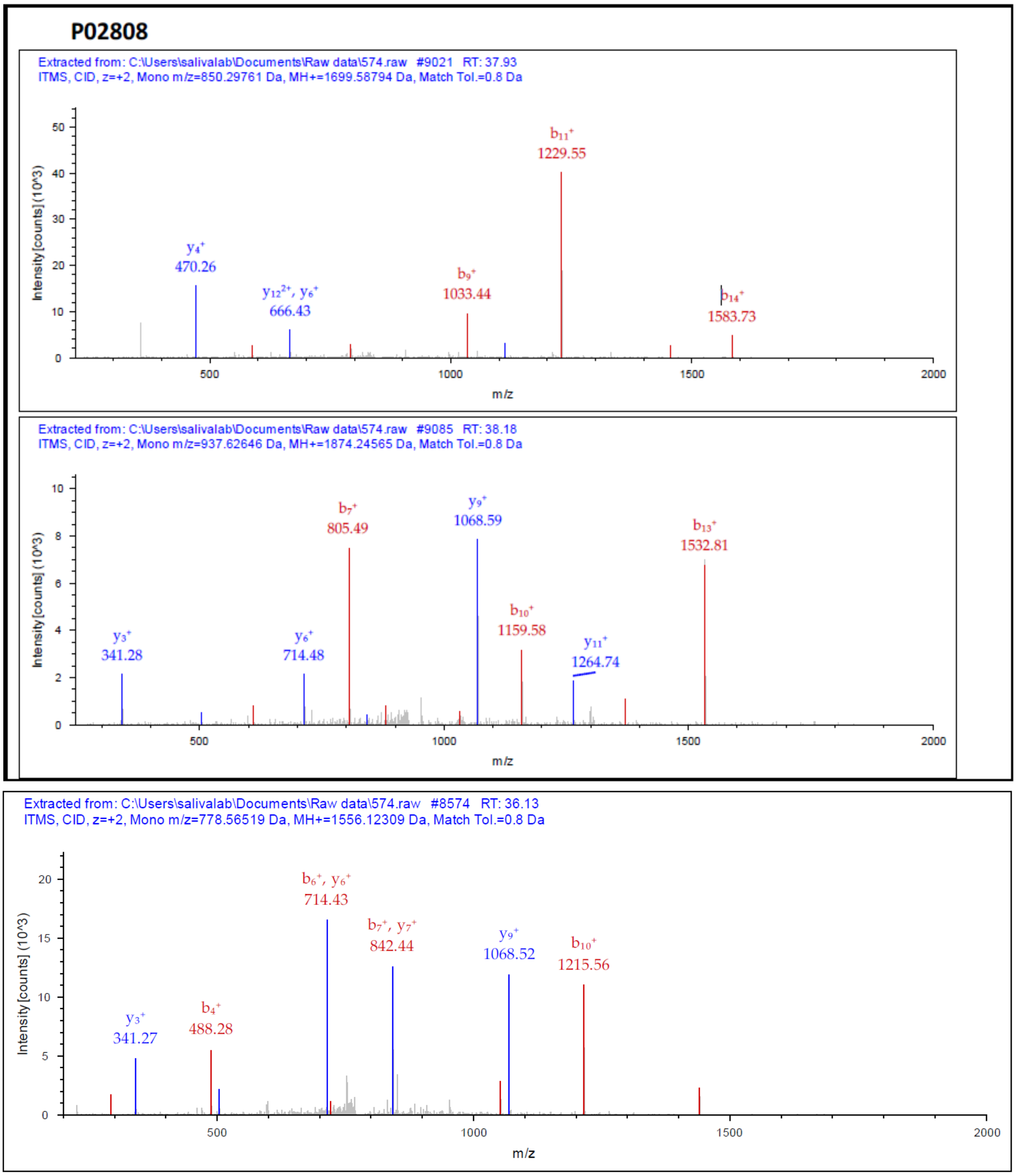

Figure S2. Identified MS/MS spectra for naturally occurring AEP peptides for Salivary acidic proline-rich phosphoprotein 1/2 (P02810).

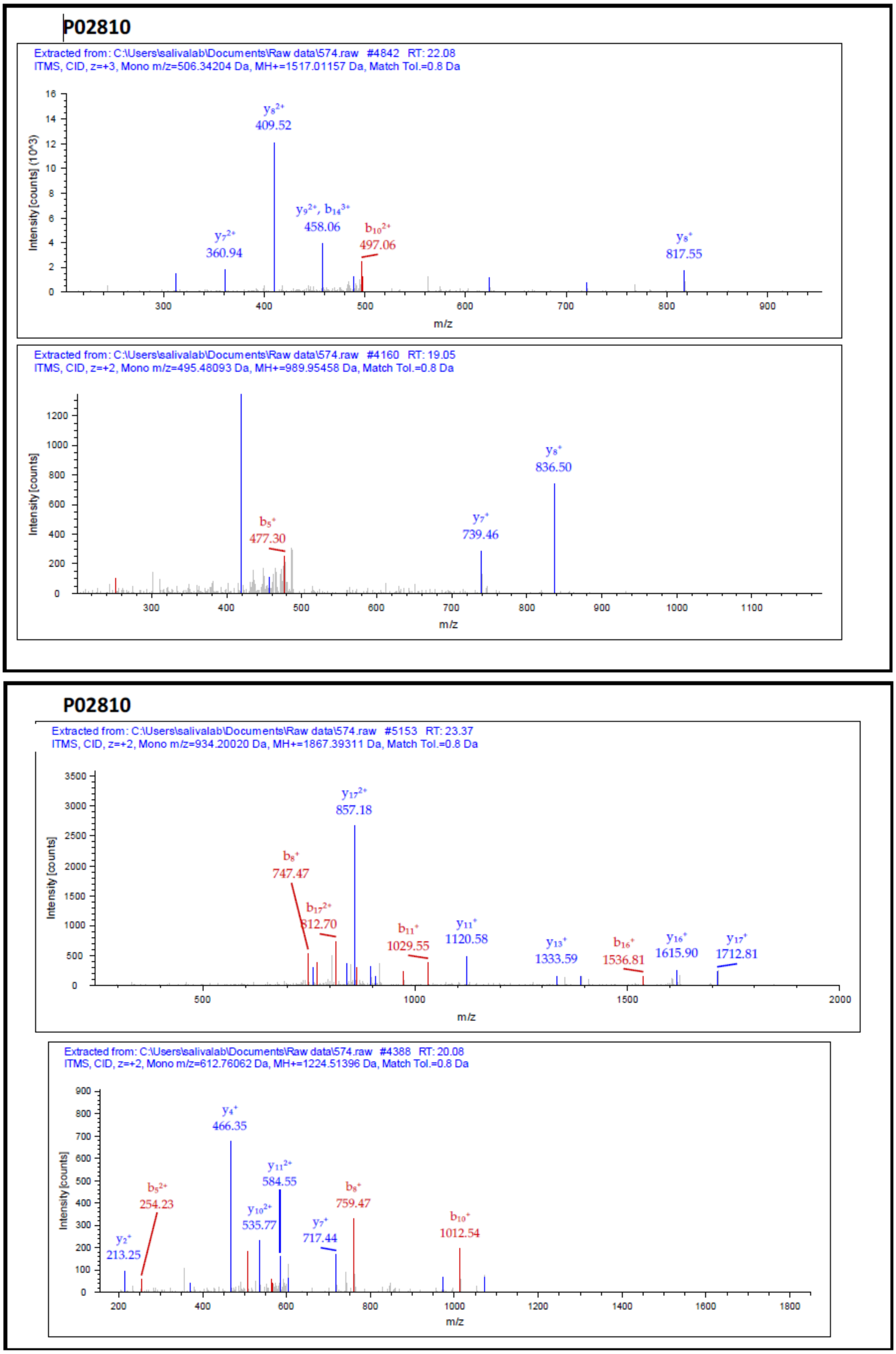

Figure S2. *Cont.*

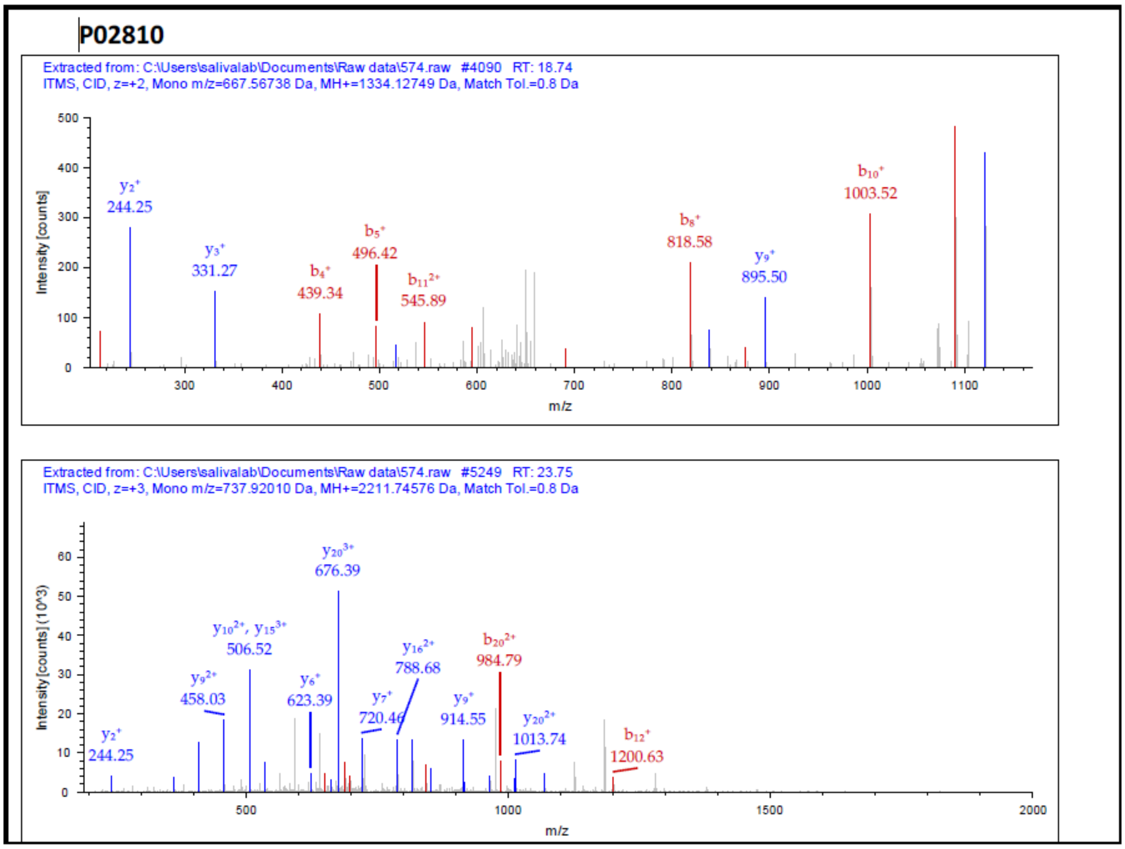

Figure S3. Identified MS/MS spectra for naturally occurring AEP peptides for Basic salivary proline-rich protein 2 (P02812).

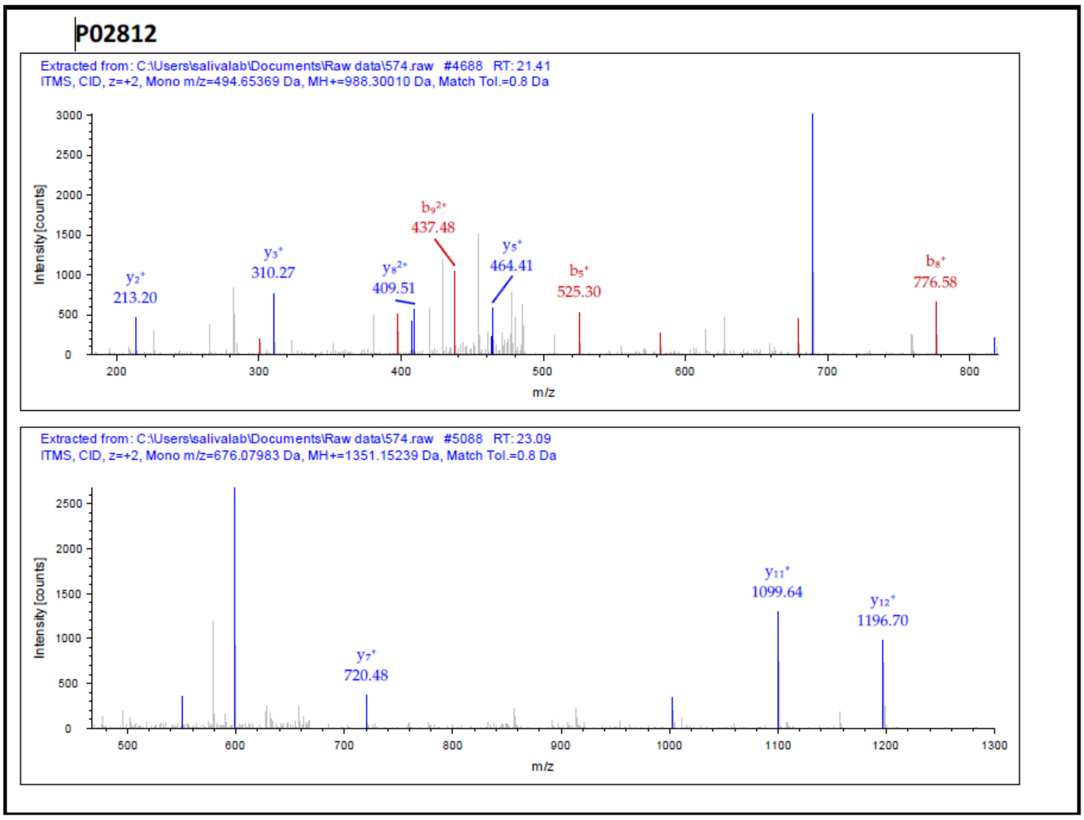

Figure S3. *Cont.*

P02812

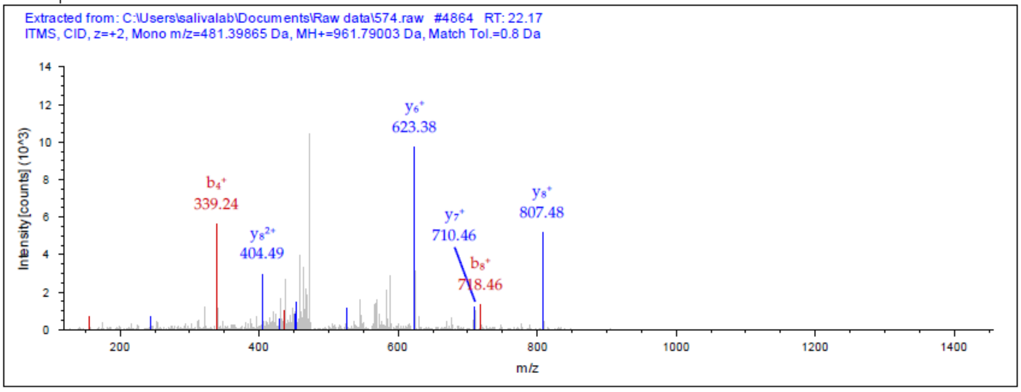

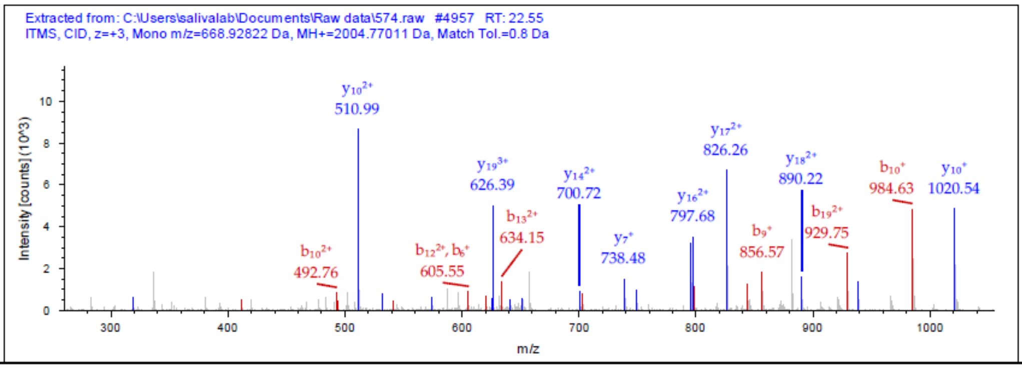

P02812

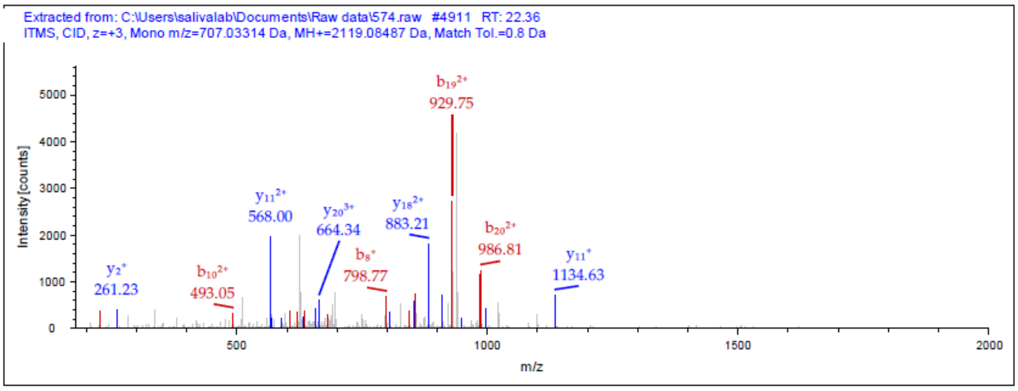

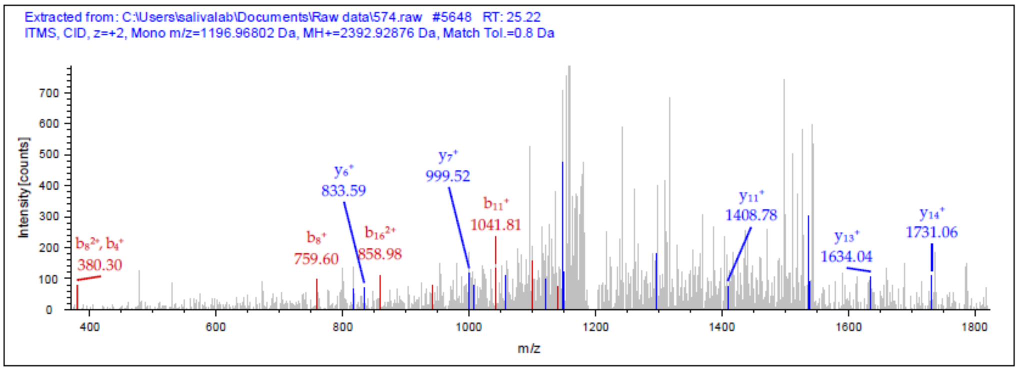

Figure S3. *Cont.*

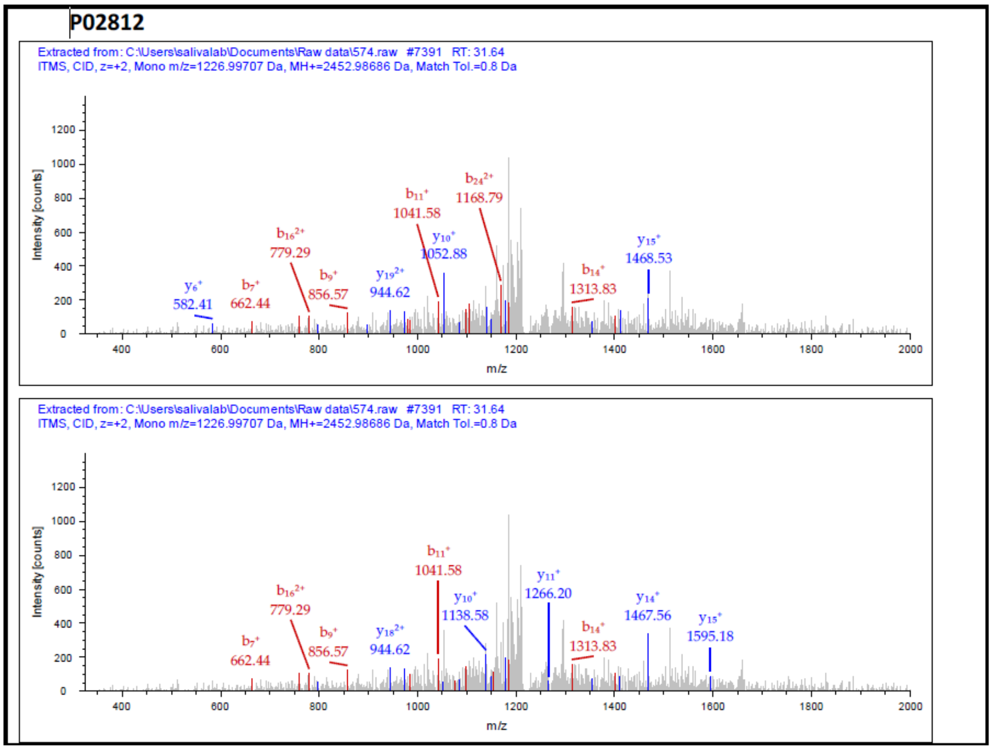

Figure S4. Identified MS/MS spectra for naturally occurring AEP peptides for Submaxillary gland androgen-regulated protein 3 (P02814).

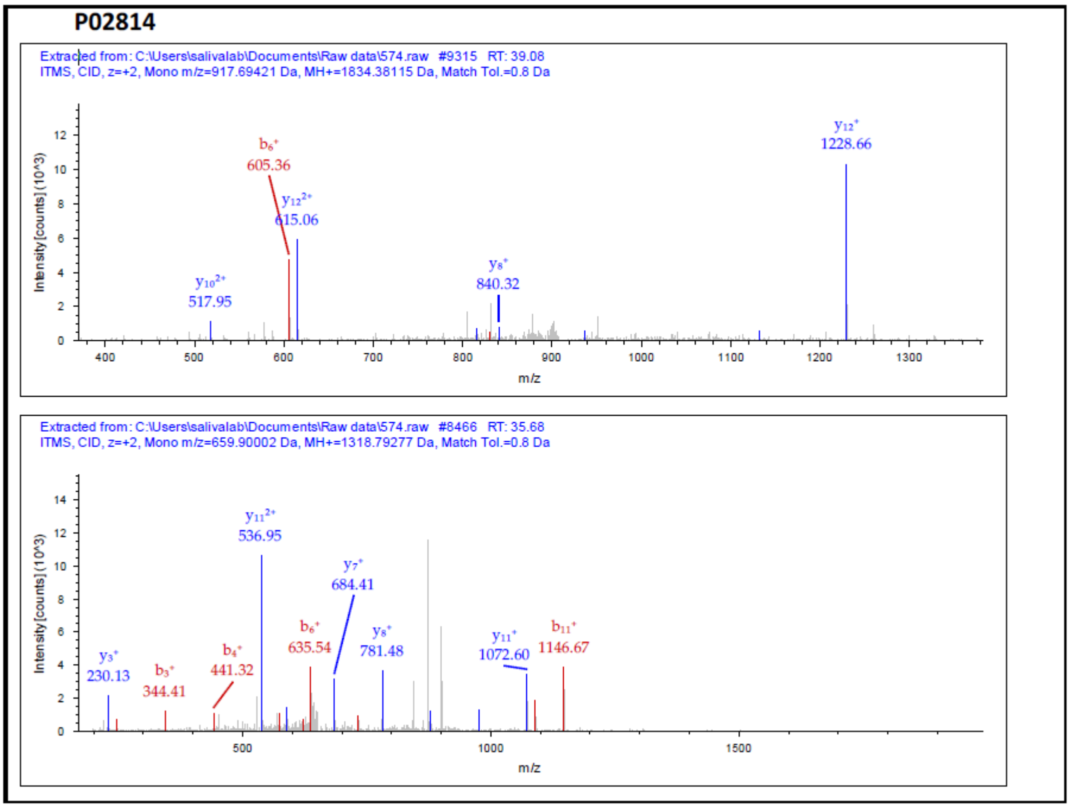

Figure S4. *Cont.*

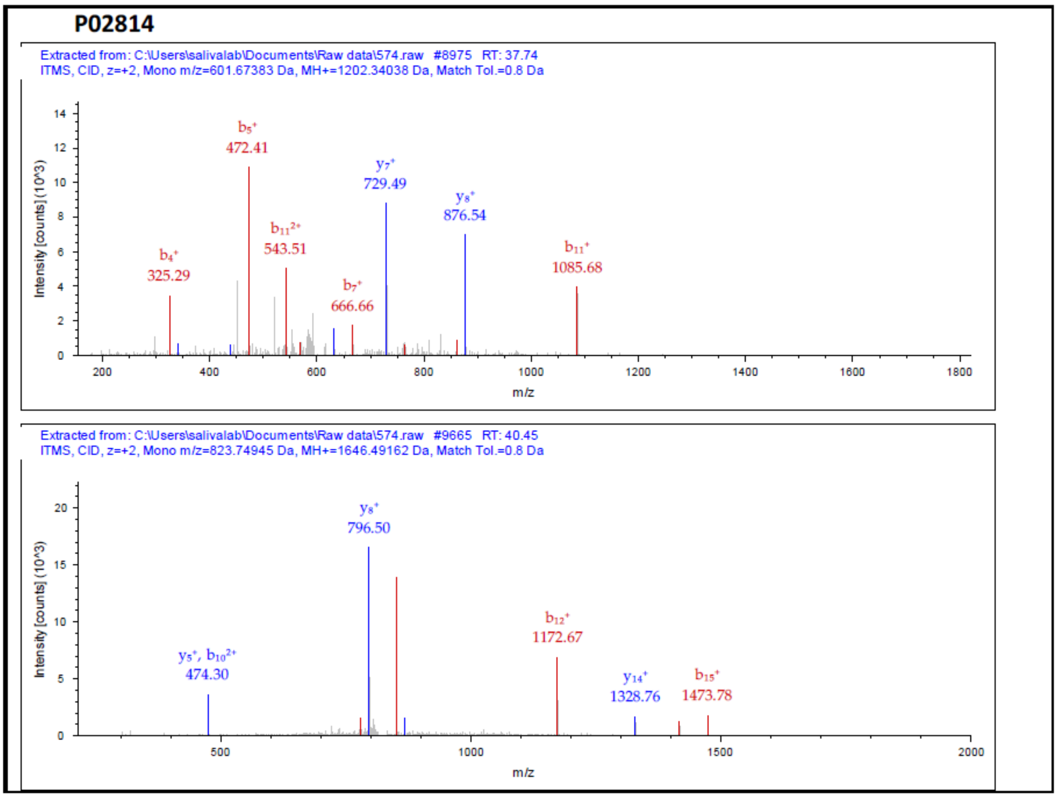

Figure S5. Identified MS/MS spectra for naturally occurring AEP peptide for cystatin-B (P04080).

P04080

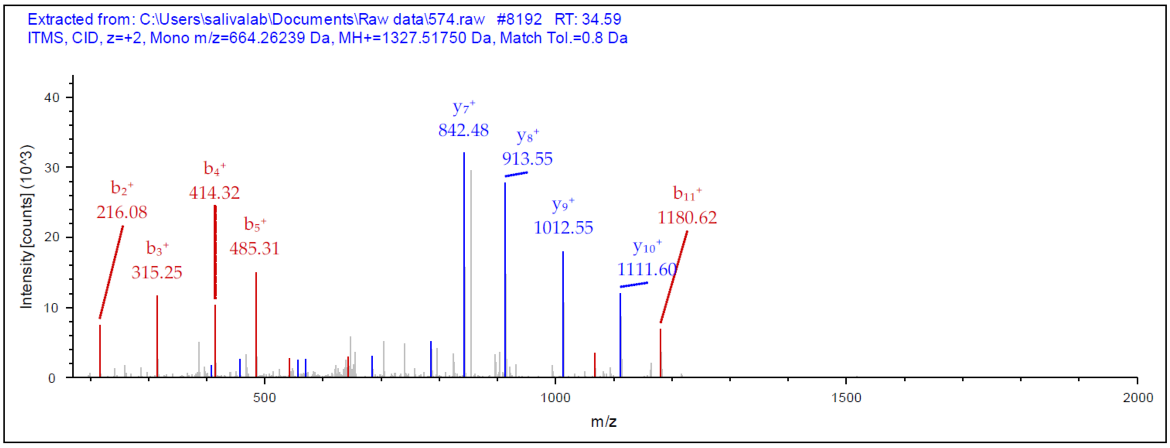

Figure S6. Identified MS/MS spectra for naturally occurring AEP peptides for Basic salivary proline-rich protein 1 (P04280).

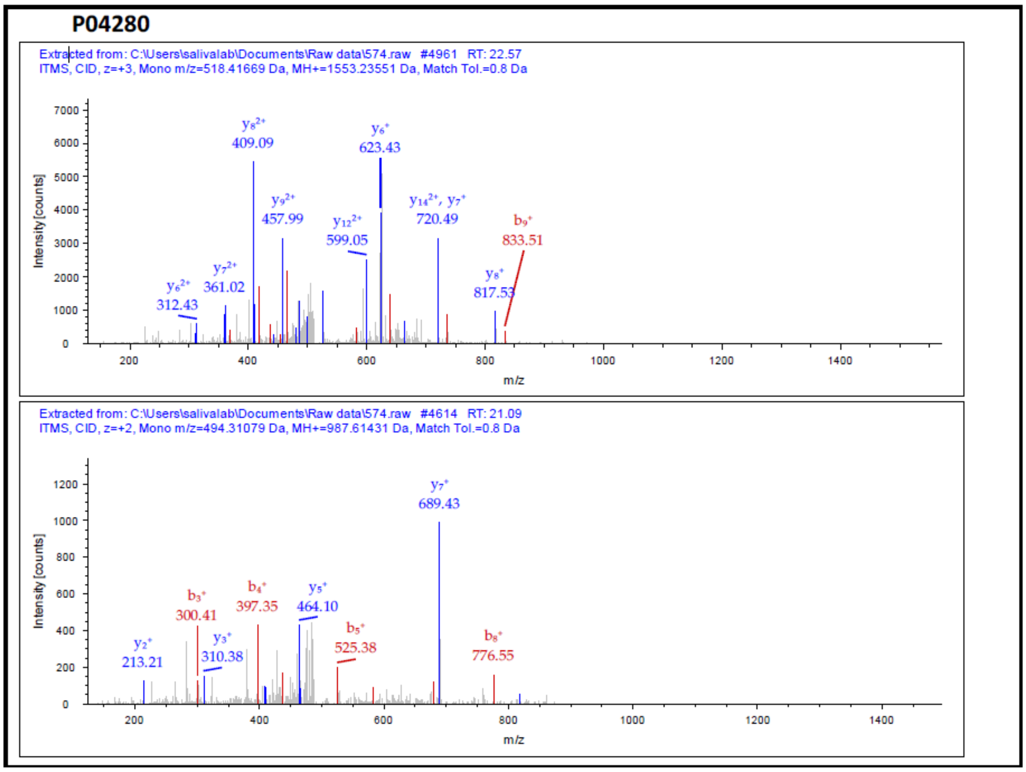

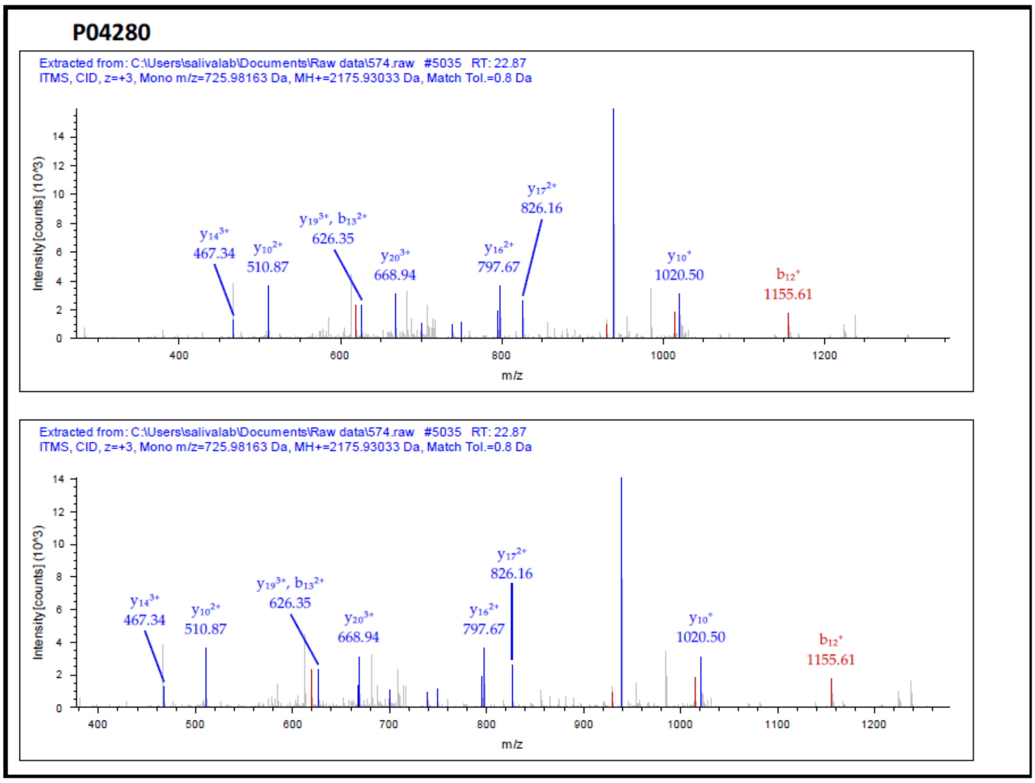

Figure S6. *Cont.*

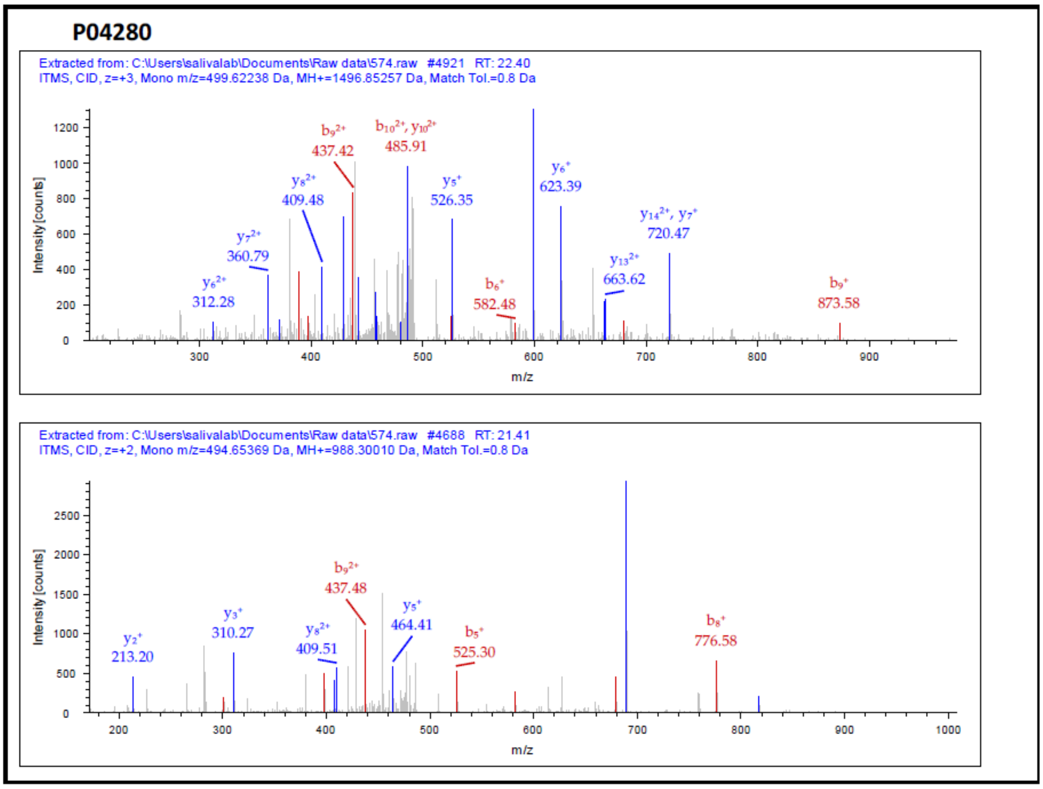

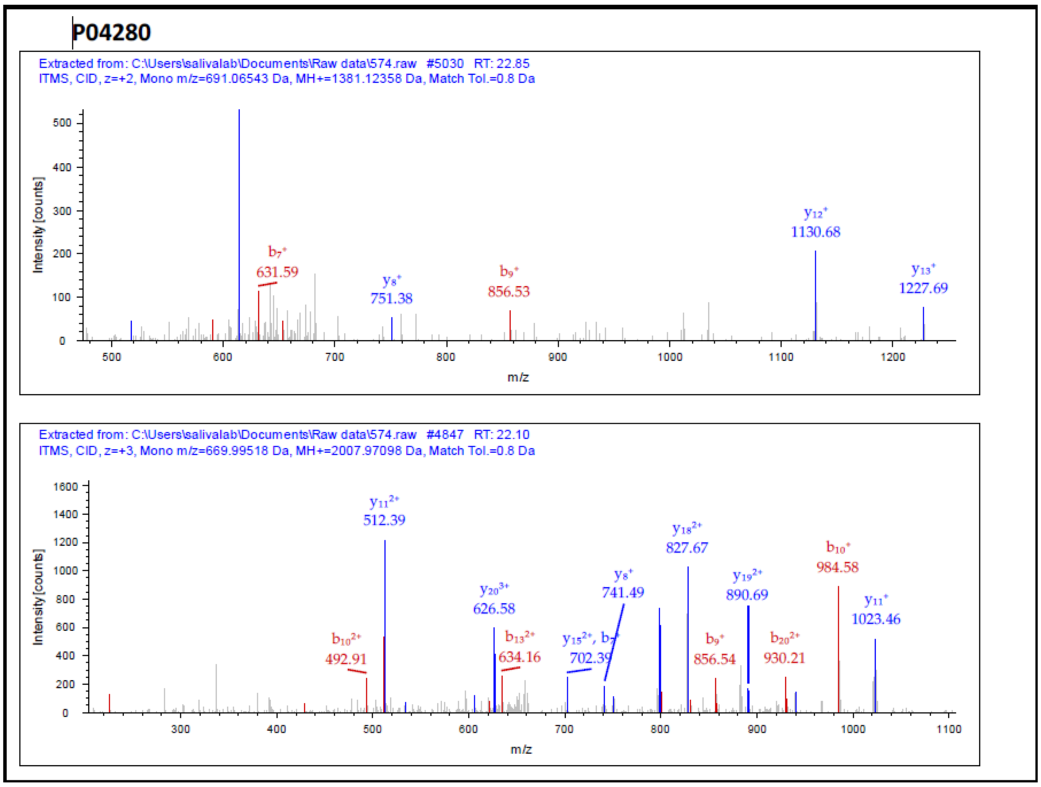

Figure S6. *Cont.*

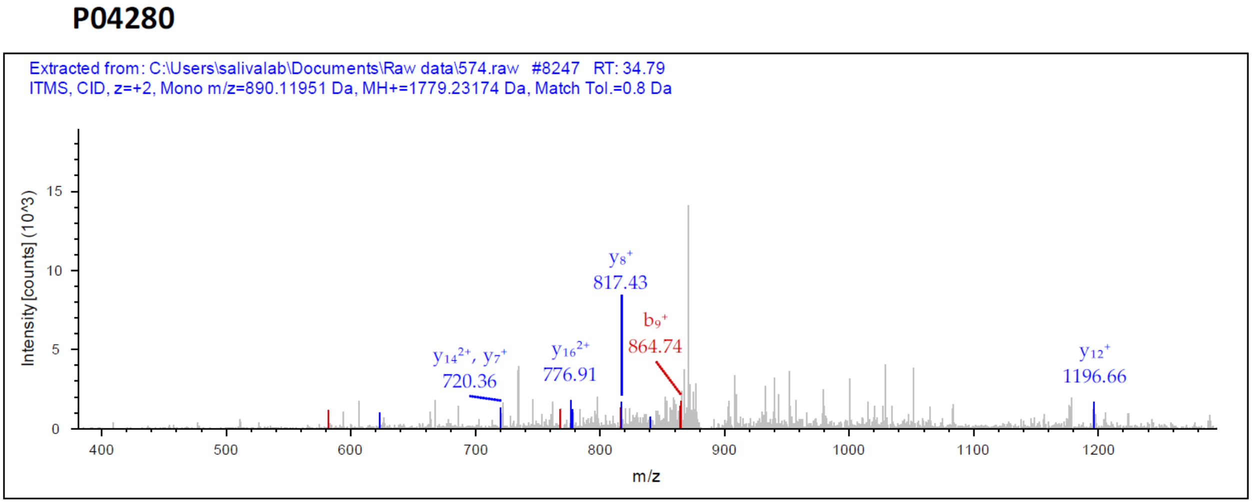

Figure S7. Identified MS/MS spectra for naturally occurring AEP peptides for S100-A9 (P06702).

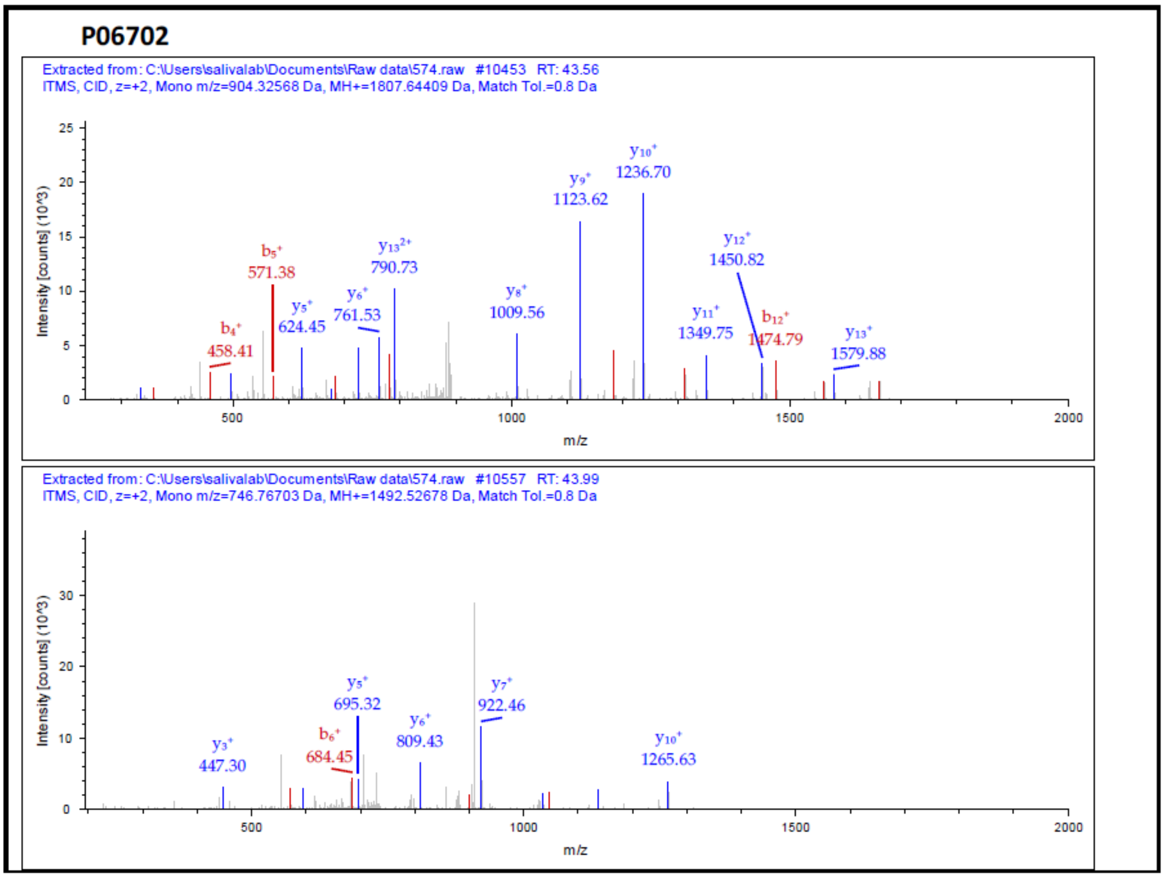

Figure S8. Identified MS/MS spectra for naturally occurring AEP peptides for Basic salivary proline-rich protein 4 allele S (P10163).

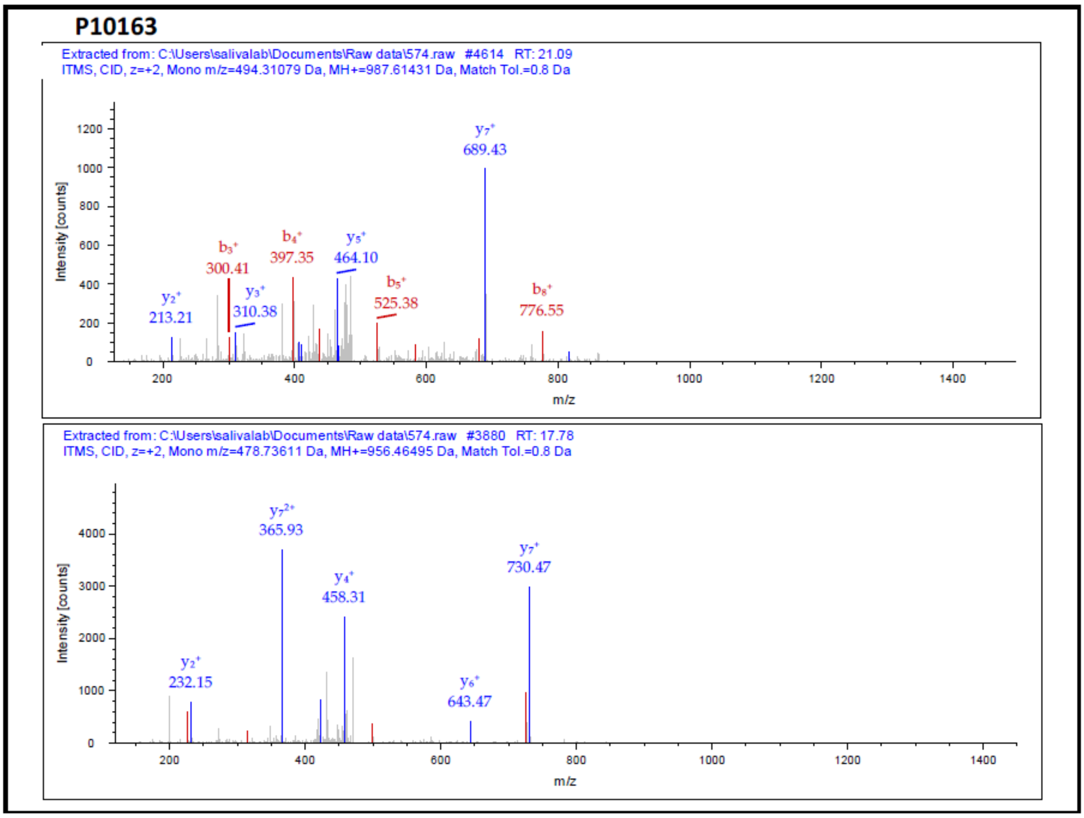

Figure S9. Identified MS/MS spectra for naturally occurring AEP peptides for Histatin 1 (P15515).

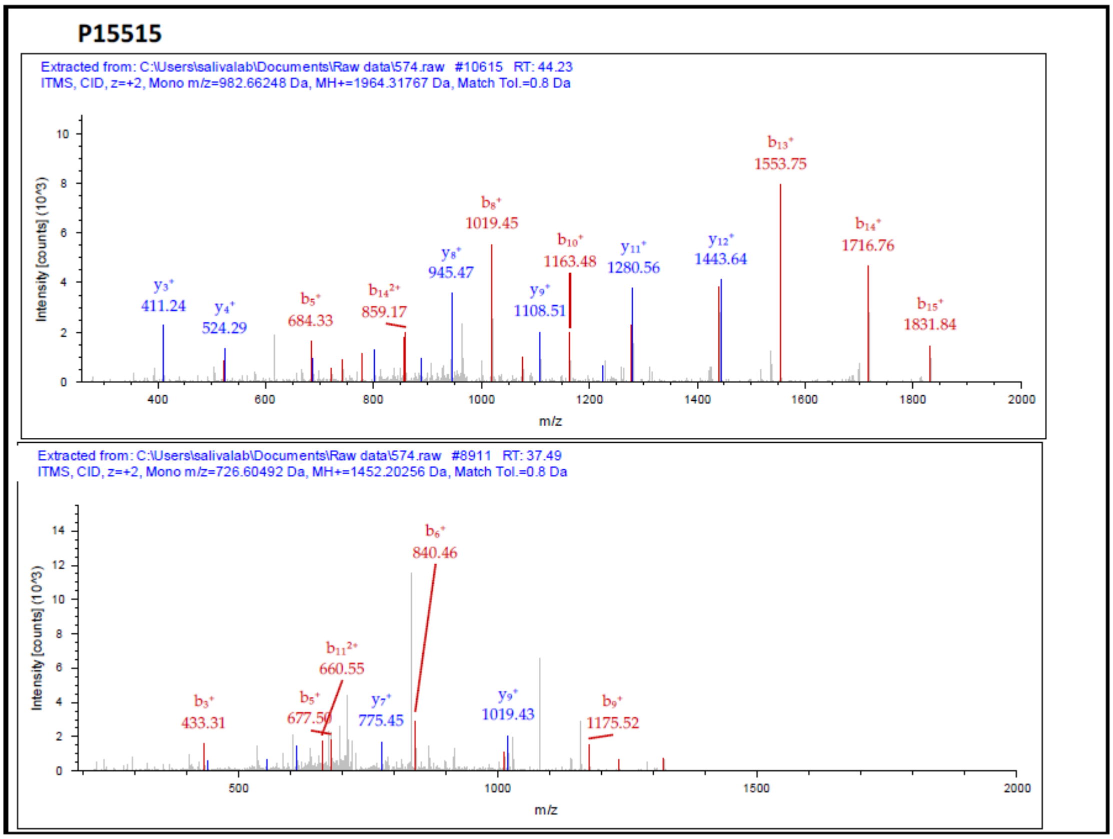

Figure S10. Identified MS/MS spectra for naturally occurring AEP peptides for Mucin-7 (Q8TAX7).

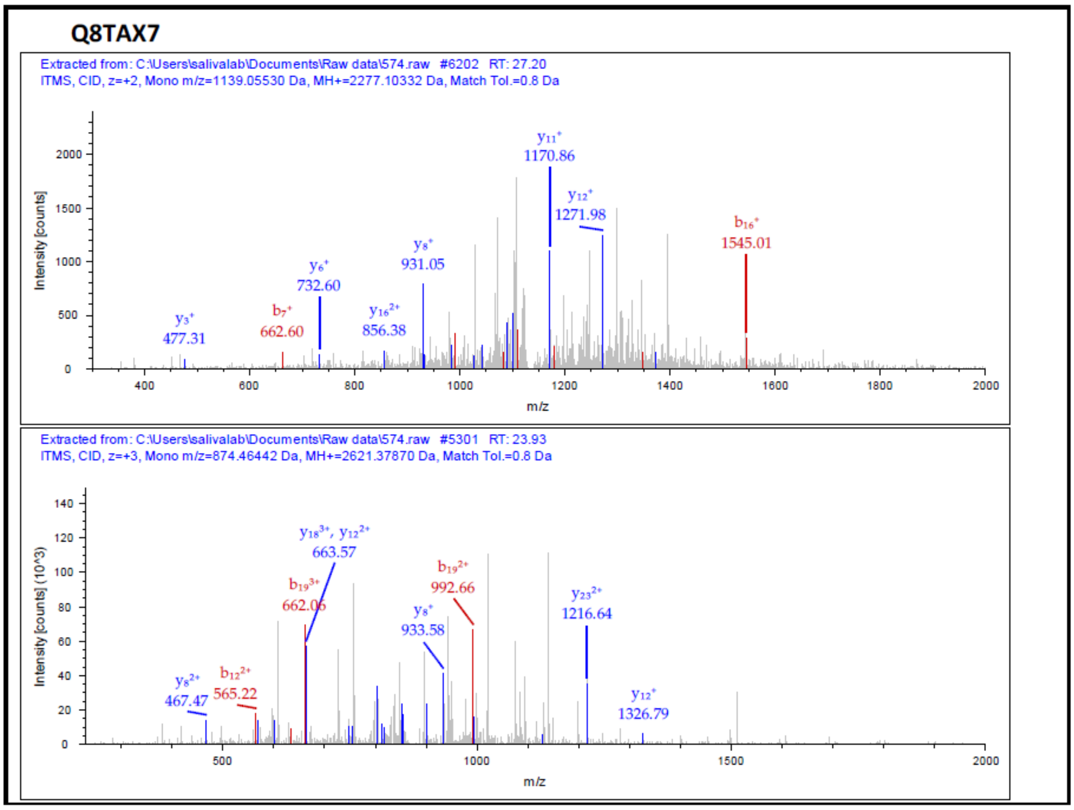

© 2013 by the authors; licensee MDPI, Basel, Switzerland. This article is an open access article distributed under the terms and conditions of the Creative Commons Attribution license (http://creativecommons.org/licenses/by/3.0/).
